# Vocalisations of Killer Whales (*Orcinus orca*) in the Bremer Canyon, Western Australia

**DOI:** 10.1371/journal.pone.0136535

**Published:** 2015-09-09

**Authors:** Rebecca Wellard, Christine Erbe, Leila Fouda, Michelle Blewitt

**Affiliations:** 1 Centre for Marine Science & Technology, Curtin University, GPO Box U1987, Perth, WA, 6845, Australia; 2 Marine Environmental Research Consultants, Sydney, NSW, 2099, Australia; 3 University of Sydney Institute of Marine Science, University of Sydney, Sydney, NSW, 2006, Australia; Aristotle University of Thessaloniki, GREECE

## Abstract

To date, there has been no dedicated study in Australian waters on the acoustics of killer whales. Hence no information has been published on the sounds produced by killer whales from this region. Here we present the first acoustical analysis of recordings collected off the Western Australian coast. Underwater sounds produced by Australian killer whales were recorded during the months of February and March 2014 and 2015 in the Bremer Canyon in Western Australia. Vocalisations recorded included echolocation clicks, burst-pulse sounds and whistles. A total of 28 hours and 29 minutes were recorded and analysed, with 2376 killer whale calls (whistles and burst-pulse sounds) detected. Recordings of poor quality or signal-to-noise ratio were excluded from analysis, resulting in 142 whistles and burst-pulse vocalisations suitable for analysis and categorisation. These were grouped based on their spectrographic features into nine Bremer Canyon (BC) “call types”. The frequency of the fundamental contours of all call types ranged from 600 Hz to 29 kHz. Calls ranged from 0.05 to 11.3 seconds in duration. Biosonar clicks were also recorded, but not studied further. Surface behaviours noted during acoustic recordings were categorised as either travelling or social behaviour. A detailed description of the acoustic characteristics is necessary for species acoustic identification and for the development of passive acoustic tools for population monitoring, including assessments of population status, habitat usage, migration patterns, behaviour and acoustic ecology. This study provides the first quantitative assessment and report on the acoustic features of killer whales vocalisations in Australian waters, and presents an opportunity to further investigate this little-known population.

## Introduction

The killer whale (*Orcinus orca)* is a cosmopolitan marine mammal found in all oceans of the world [1]. Currently considered one species, different populations of killer whales can be categorised into distinct ‘ecotypes’, based on substantial differences in morphology, behaviour, diet and acoustic repertoire. Sympatric ecotype assemblages are currently documented from three different geographical regions: the eastern North Atlantic, the eastern North Pacific and Antarctica [2–5].

In Antarctic waters, three different morphological forms (morphotypes) of killer whales were originally identified, with differences in the suggested ecological specialisations possibly being even more pronounced than those reported for the eastern North Pacific ecotypes [3]. Further research to date describes five distinct killer whale morphotypes in Antarctic waters [Types A, B (two forms), C and sub-Antarctic Type D], each with their own physiological, morphological and social adaptations [6].

In Australia, killer whales have been sighted in all state and territory waters [7–9]. Nonetheless, no defined killer whale ecotypes have been described in Australian waters due to limited knowledge of their distribution, movements, habitat use and population status. To date, there has been no reliable estimate of the population size of killer whales in Australian waters, and population trends are unknown, with much of the information on killer whale distribution and occurrence obtained from incidental sightings, and from one sighting program undertaken on Macquarie Island [10]. Notably, they are more commonly sighted in coastal waters, along the continental shelf around south-eastern Tasmania, Victoria and southern New South Wales, around sub-Antarctic Macquarie Island, and in some parts of the Australian Antarctic Territory [7–9, 11–19]. The limited knowledge of the spatial and temporal extent of killer whale movements throughout the Australian region means dedicated surveys of killer whales are required to quantify their distribution, movements, habitat use, population size and trends.

Acoustic communication is widely utilised by cetaceans in a range of contexts, including social interactions, group cohesion, mating, mother-calf contact, travelling and foraging [20]. In addition, odontocetes use echolocation during navigation and hunting [21]. Research from the northern hemisphere has demonstrated that killer whales predominantly produce three commonly grouped sounds: echolocation clicks, burst-pulse sounds and whistles.

Echolocation clicks are short-duration (< 250 μs), broadband (10 kHz– 100 kHz) pulses of up to 224 dB re 1 μPa @ 1 m peak-to-peak source level, typically emitted in trains with a several-second duration [4, 22–26]. Whistles and burst-pulse sounds are thought to be communicative signals most commonly used in social contexts [23, 27]. Whistles are frequency-modulated, tonal sounds, with or without harmonic overtones, with the fundamental frequency ranging from 1 to 36 kHz and source levels up to 193 dB re 1 μPa @ 1 m peak-to-peak recorded from the North Pacific killer whale populations [23, 28–31], and fundamental frequencies up to 74 kHz recorded in Norwegian and Icelandic killer whales [32]. Burst-pulse sounds consist of rapidly repeated pulses with inter-pulse intervals shorter than in echolocation click trains, and are considered to function as contact signals in group recognition and coordination of behaviour [23, 33]. In spectrographic images, burst-pulse sounds typically appear as frequency-modulated sounds with numerous sidebands and overtones. The energy of burst-pulse sounds usually lies between 500 Hz and 25 kHz, lasting 0.5–1.5 s, with source levels of 131–176 dB re 1 μPa @ 1 m root-mean-square [23, 26, 34–41].

Call structure varies amongst allopatric, parapatric and sympatric killer whale populations.

Differences in calls amongst spatially separated populations of killer whales are apparent from studies across the world, e.g. the North Pacific [23, 37, 42, 43], Norway [41], and Antarctica [40]. There has also been evidence of dialects amongst social groups within a population. The resident populations of western Canada and north-western USA consist of four acoustic clans, each clan containing group-specific repertoires reflecting the maternal genetic relationship of the groups [39]. Pods within a clan share call types, but exhibit pod-specific variation, i.e. dialects, of shared call types. Such group-specific dialects have also been documented in killer whale populations in Norway and Iceland [41, 44].

Passive acoustic monitoring can be an inexpensive and effective way of observing cetacean distribution, migration, behaviour and population density [45]. However, no information has been published on the sounds produced by killer whales from the Australian region. A detailed description of the acoustic characteristics is necessary for species acoustic identification, as well as instituting a basis for comparison of the acoustics of other killer whale populations worldwide and uncovering potential distinctive repertoires in the Australian population. Bioacoustics can also aid in identifying potential sympatric ecotypes in Australia.

Here we present results of the first quantitative study on the acoustic features of underwater sounds produced by killer whales in Australian waters.

## Materials and Methods

### Data Collection

Non-systematic surveys were conducted in the Bremer Canyon in southern Western Australia, between February and March 2014 and again in February and March 2015 (study area within 20 nautical mile radius of centre point: 34°44.30'S latitude and 119°35.55'E longitude). Data was collected from two vessels, a research vessel and a commercial ecotourism vessel, during daylight hours and variable weather conditions. A total of 34 field trips were conducted with more than 278 hours spent at sea, resulting in 85 encounters with killer whales.

Upon an encounter with a pod, information on the group composition, number of animals and behavioural state was recorded. Surface behaviour was assigned to one of four behavioural states, which were modelled from previous killer whale studies [4, 23, 46, 47]: (1) travelling, (2) feeding, (3) milling and (4) socialising. Identification photographs of each pod encountered were collected following established methodologies for killer whale photo-identification studies [48, 49].

Acoustic recordings were obtained with two devices. Primarily recordings were undertaken with a High Tech Inc. HTI-96-MIN hydrophone with built-in pre-amplifier (flat frequency response of 2 Hz to 30 kHz; sensitivity -164.1 dB re 1 V/μPa), fitted to a Sound Devices 722 digital recorder sampling at 96 kHz, 24-bit. Recordings were opportunistic, and once the vessel was manoeuvred into a close proximity of no less than 100 m to the focal group, the engine was switched off and the hydrophone was deployed over the side of the vessel, suspended from a buoy by a bungee including a damper, and lowered down to 5 m below the sea surface using a small weight. Secondary to this, a SoundTrap (Ocean Instruments, New Zealand)—a self-contained underwater sound recorder—was rigged to a tow-line and deployed overside during an encounter when the vessel was travelling less than 5 knots. The SoundTrap sampled at 192 kHz, 16-bit.

### Data Analysis

Acoustic recordings were downloaded from the Sound Devices 722 digital recorder and SoundTrap unit onto a computer and inspected both visually and aurally using acoustic software Raven Pro 1.5 [50]. Calls with a good signal-to-noise ratio (SNR) were selected and analysed using custom software written in MATLAB [51]. A Hamming window was used to compute spectrograms with 50% overlap. Only recordings made during confirmed visual species sighting were included in analysis. All data where another distinguishable or undistinguishable odontocete species was observed within or near the pod of recording was excluded from analysis.

We grouped all sounds into whistles, burst-pulse sounds and clicks. We did not perform any quantitative analysis of the clicks, as these are not expected to be population-specific or characteristic, but focussed on whistles and burst-pulse sounds for this study. We defined whistles as continuous, frequency-modulated, tonal sounds consisting of the fundamental frequency, and in some cases with harmonic overtones at frequencies that were integer multiples of the fundamental. Burst-pulse sounds, on the other hand, consisted of rapidly repeated broadband pulses and appeared in spectrograms as constant-wave or frequency-modulated contours with many sidebands and overtones.

**Fig 1:**
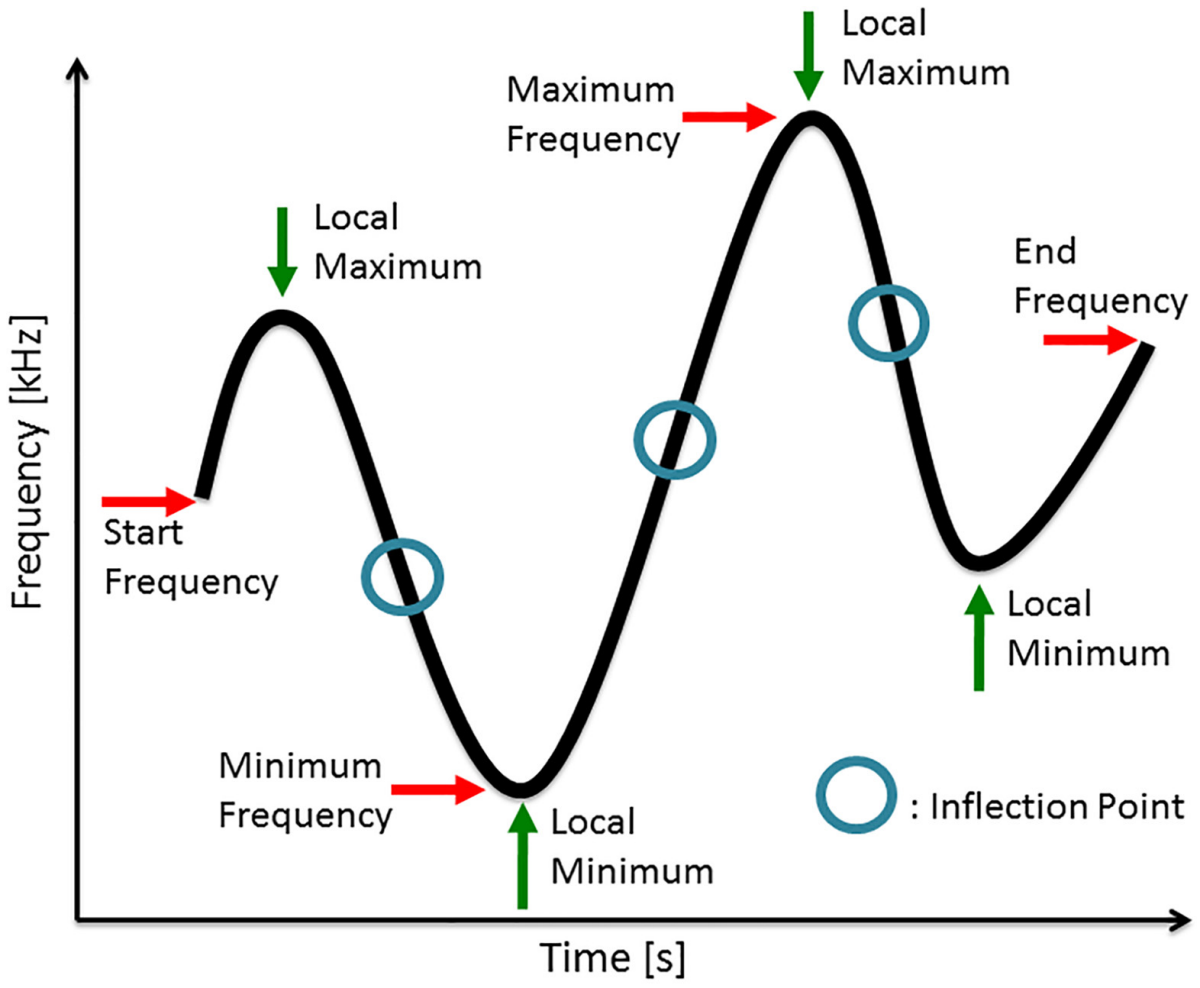
Parameters that are measured for sound analysis of killer whale vocalisations.

For each whistle, we measured the following parameters of its fundamental: minimum frequency (Min f), maximum frequency (Max f), range in frequency (Delta f, i.e. Max f − Min f), start frequency (Start f), end frequency (End f), duration, number of extrema, number of inflection points, and frequency modulation rate (FM rate). We further determined whether there were any harmonically related overtones or not. Extrema are local maxima and local minima in the fundamental, i.e. stationary points, where the first derivative (the slope) is 0 and changes sign (from positive to negative in the case of a local maximum and from negative to positive in the case of a local minimum). At inflection points, the fundamental changes curvature, and the first derivate has a local extremum. FM rate was computed as the ratio of the number of inflection points and the duration (Fig 1).

In the case of burst-pulse sounds, we measured the minimum frequency (Min f) and the maximum frequency (Max f) of the lowest contour, its start frequency (Start f) and end frequency (End f), its range in frequency (Delta f), duration, number of extrema, number of inflection points, frequency modulation rate (FM rate), and the sideband spacing of the burst-pulse sound.

### Statistical Analysis

The parameters measured and described as above made up a feature vector for each call. K-means clustering [52], a simplification of Gaussian mixture modelling, was applied to group the calls into categories by minimising the Euclidian distance between all feature vectors and the cluster centroids. We performed this analysis in MATLAB [51], using the k-means algorithm of the MATLAB statistics toolbox.

## Results

A total of 28 hours and 29 minutes of underwater recordings was examined with 2376 orca vocalisations detected. Vocalisations with poor SNR were excluded from analysis, resulting in 142 vocalisations suitable for analysis and categorisation, with all groupings presented in this paper.

**Fig 2:**
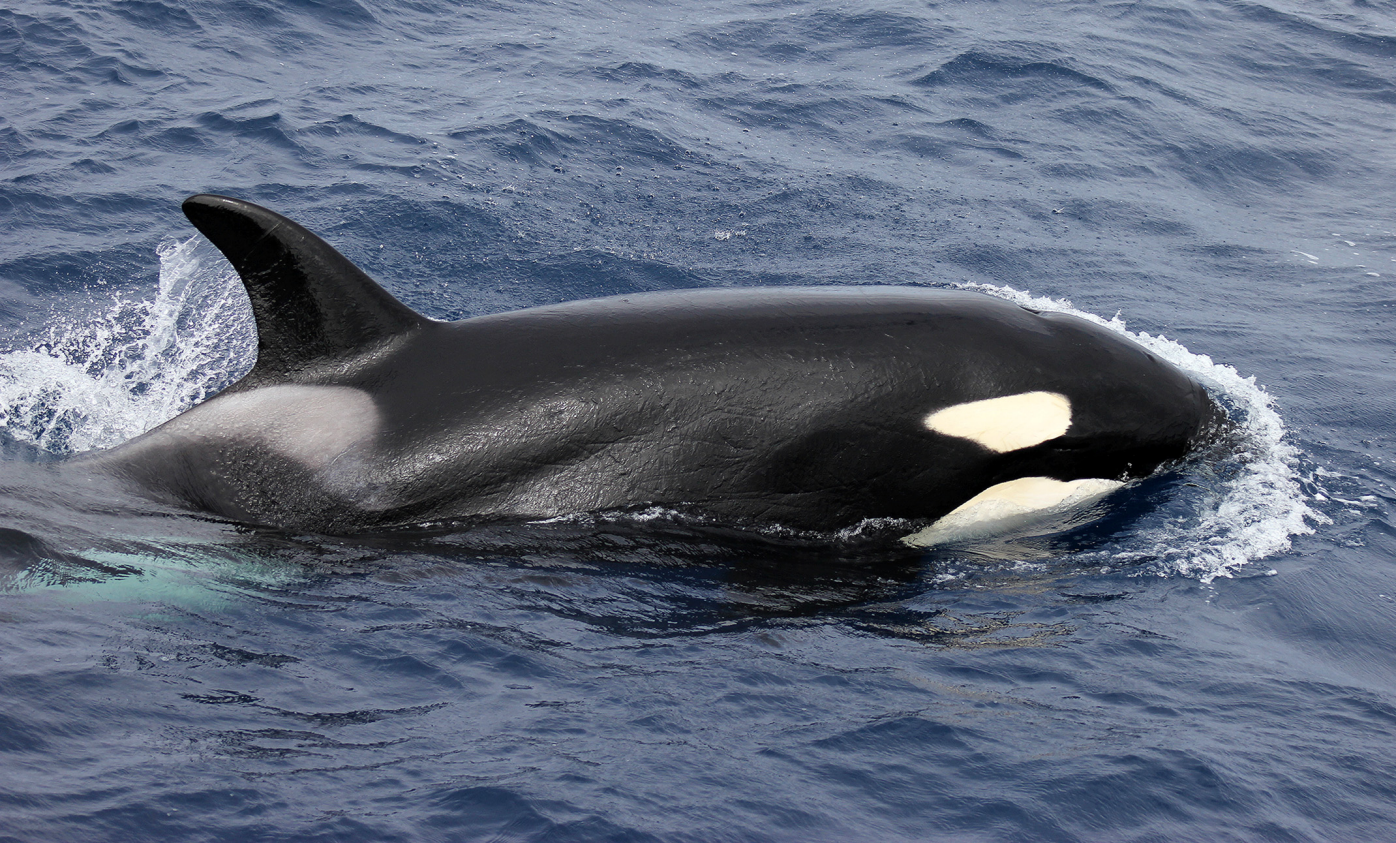
Animal sighted during acoustic recordings at field site in Western Australia.

**Fig 3:**
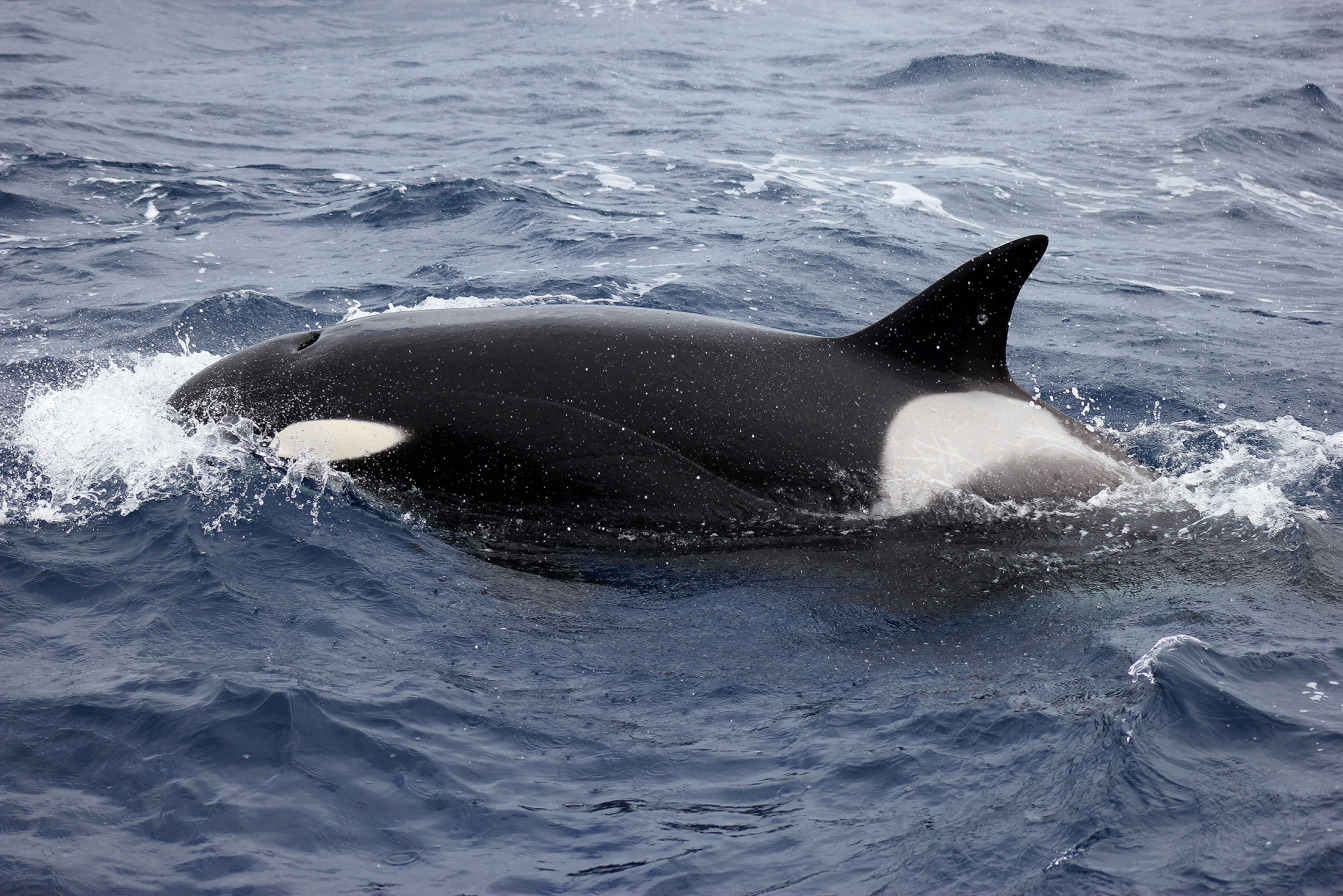
Animal sighted during acoustic recordings at field site in Western Australia.

Due to the limited knowledge of Australian killer whale distribution, movements, habitat use and populations, no known ecotypes have been identified or described for the Australian region. Animals sighted during recordings displayed phenotypic characteristics concurrent with ecotype Type A as described by Pitman and Ensor [3] (see Figs 2 and 3). Although it must be noted, categorizing these animals into such ‘ecotypes’ should be with caution, since Type A is described for animals specifically sighted in the Antarctic region.

All behaviours documented during acoustic recordings were categorised as either travelling or social behaviour. No acoustic recordings were made whilst observing feeding or milling behaviour.

The following figures illustrate spectrographic examples of the various call types. Table 1 summarises the measurements of whistles, and Table 2 summarises the measurements of burst-pulse sounds and remaining groups.

**Table 1:** Summary of measurements for categorized whistles produced by killer whales recorded in Bremer Canyon, Western Australia.

GROUP	N	Min f (kHz)	Max f (kHz)	Start f (kHz)	End f (kHz)	Delta f (kHz)	Duration (s)	Number of extrema	Number of inflection points	FM rate (1/s)
**BC01**	18	2.2–11.8	3.9–14.6	2.2–12.8	2.9–14.5	1.1–6.2	0.2–3.7	9–21	8–20	3.4–50.2
**BC02**	35	1.0–5.4	1.8–8.4	1.1–6.3	1.0–8.4	0.4–4.4	0.1–1.5	0–9	0–8	0–38
**BC03**	5	6.8–8.2	10.0–11.5	7.6–8.4	7.5–9.7	3.2–3.8	3.9–11.3	15–72	14–71	3.3–6.3
**BC04**	61	3.9–15.0	9.1–29.3	3.9–27	5.7–29.1	0.3–20.3	0.05–1.4	0–7	0–6	0–14.3

Number of whistles per group: n. For each group, the range over all of the calls belonging to that group is given. Min f: minimum frequency of the fundamental; Max f: maximum frequency of the fundamental; Start f: frequency at which the fundamental commenced; End f: frequency at which the fundamental finished; Delta f = Max f − Min f; Duration (s); Number of extrema; Number of inflection points; FM rate = Number of inflections points / duration.

**Table 2:** Summary of measurements for categorized burst-pulse sounds and remaining groups produced by killer whales recorded in Bremer Canyon, Western Australia.

GROUP	N	Min f (kHz)	Max f (kHz)	Start f (kHz)	End f (kHz)	Delta f (kHz)	Duration (s)	Number of extrema	Number of inflection points	FM rate (1/s)	Sideband spacing (kHz)
**BC05**	5	0.6–1.2	1.1–5.6	0.6–1.5	0.7–3.4	0.3–4.5	0.5–1.2	0–5	0–4	0–6.5	0.3–0.7
**BC06**	6	4.6–8.1	5.2–10.7	4.8–10.1	4.6–8.3	0.6–2.8	0.2–0.6	4–8	5–7	9.3–28.2	0.2–0.8
**BC07**	4	2.7–4.7	4.1–6.3	3.6–6.3	2.9–6.3	1.4–1.6	0.1–0.3	1–6	0–5	0–18.1	0.4–0.7
**BC08** *	2	3.6–4.2	5.1–5.2	5.4–8.7	3.9–8.0	1–1.6	0.1–0.5	1	0	0	0.8–0.9
**BC09** **	6	0.9–4.1	1.5–5.5	1.1–4.4	1.2–5.5	0.5–3.4	0.1–1.2	1–10	0–9	0–13.9	0.4–0.9

Number of burst-pulse sounds per group: n. For each group, the range over all of the calls belonging to that group is given. Min f: minimum frequency of the lowest contour; Max f: maximum frequency of the lowest contour; Start f: frequency of where the lowest contour commenced; End f: frequency of where the lowest contour finished; Delta f = Max f − Min f; Duration (s); Number of extrema; Number of inflection points; FM rate = Number of inflections points / duration.

* Whistles with pulsed middle section. Min f, Max f, Delta f were measured off the whistle fundamental. Duration, and numbers of extrema and inflection points are for the entire call. Sideband spacing was measured off the pulsed middle section.

**Burst-pulse = > whistle transitions and whistle = >burst-pulse transitions. Min f, Max f, Delta f were measured off the whistle fundamental. Duration, and numbers of extrema and inflection points are for the entire call. Sideband spacing was measured off the pulsed section.

### Whistles

#### Group BC01

**Fig 4:**
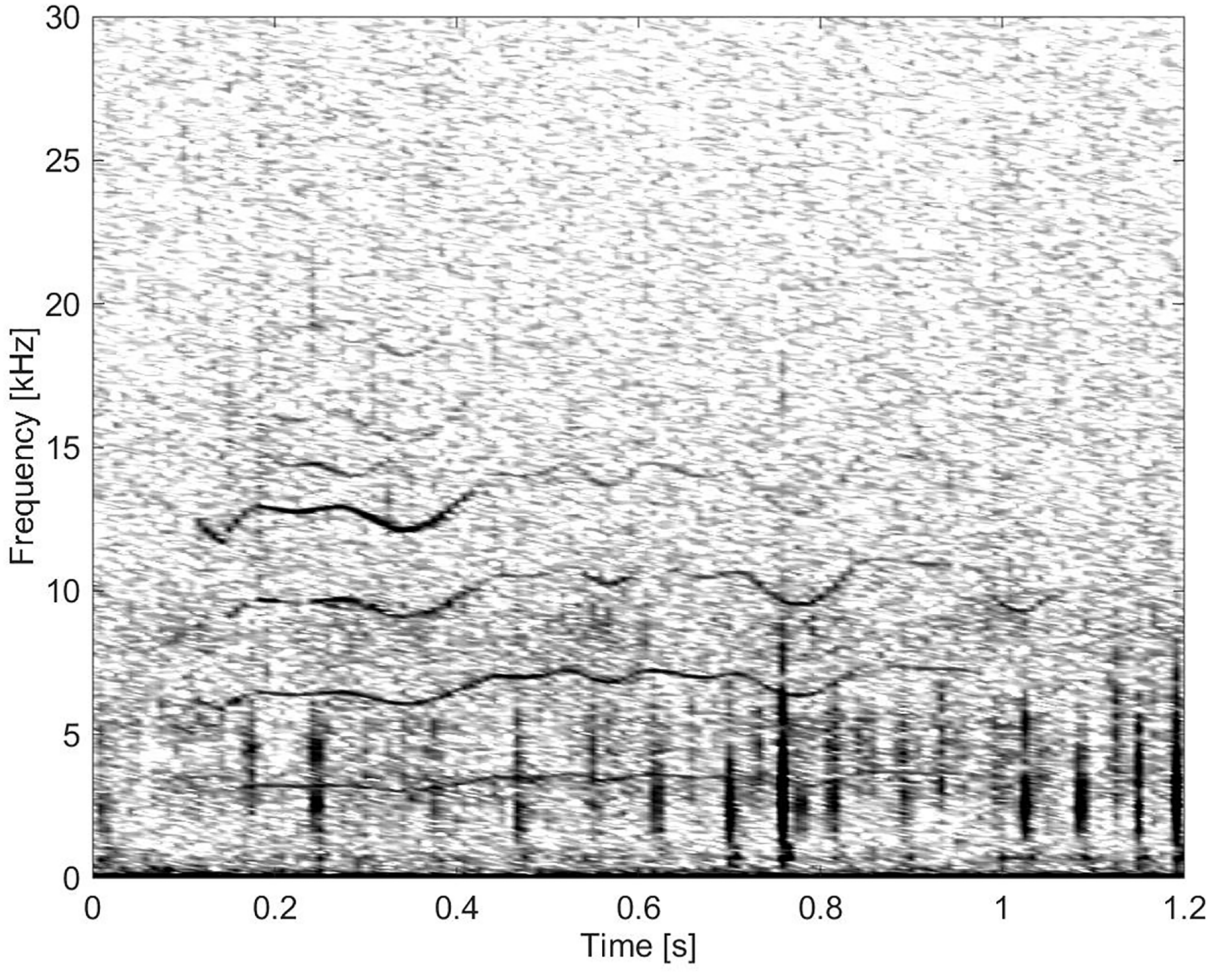
Whistle from Group BC01 with high frequency modulation (fs = 96 kHz, NFFT = 512, 50% overlap).

**Fig 5:**
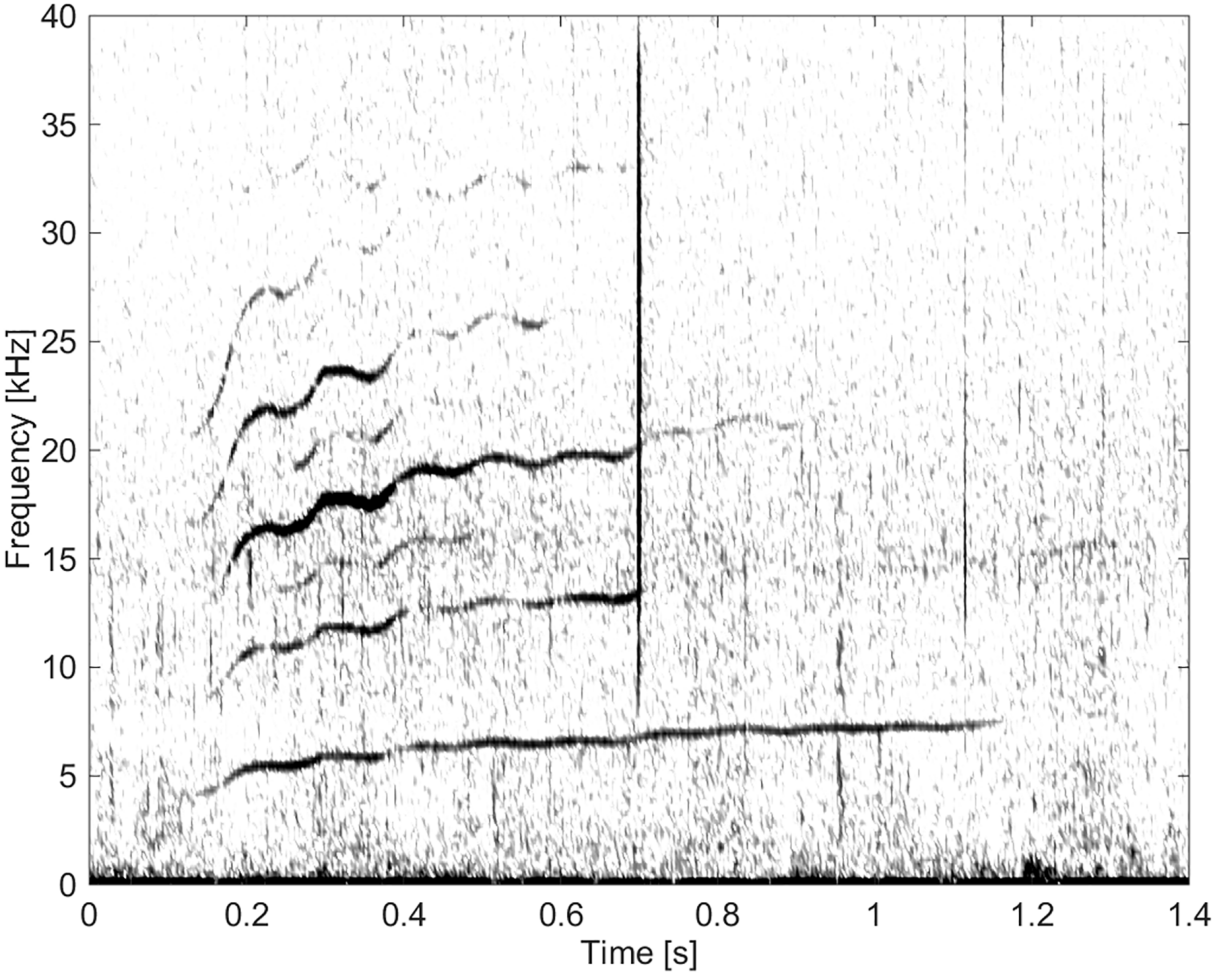
Whistle from Group BC01 with high frequency modulation and harmonic overtones. There is another faint call in the background visible at 14 kHz and 0.3 s (fs = 96 kHz, NFFT = 512, 50% overlap).

**Fig 6:**
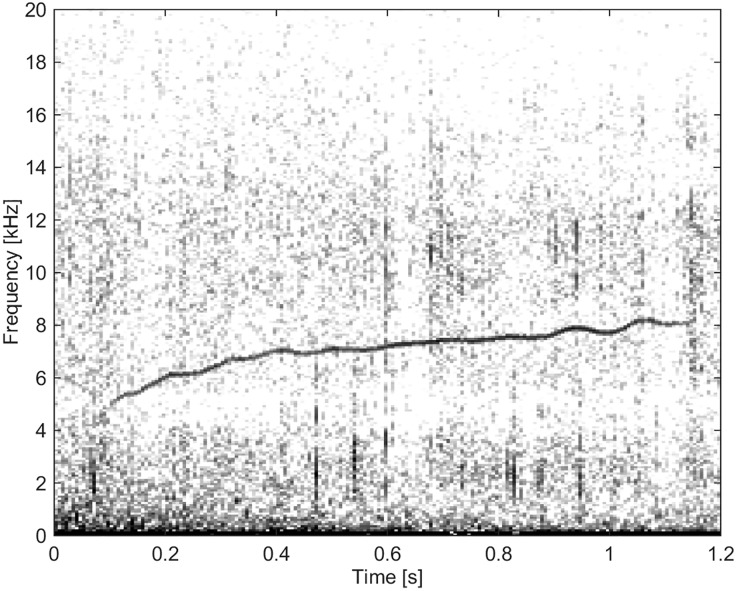
Whistle from Group BC01 with a long duration (fs = 96 kHz, NFFT = 512, 50% overlap).

**Fig 7:**
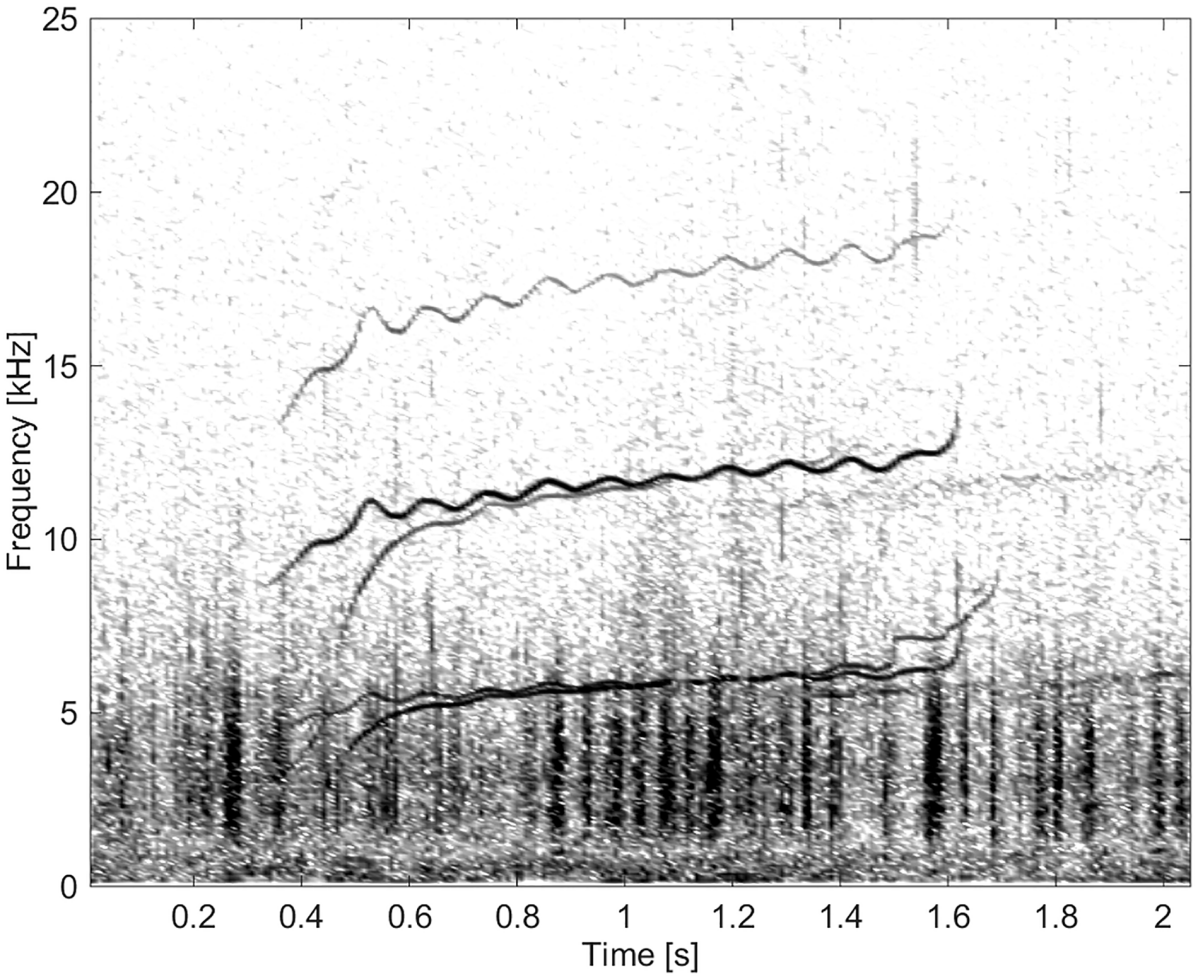
Whistle from Group BC01 with high frequency modulation and harmonic overtones. There is another call visible here which was recorded almost simultaneously as this whistle (fs = 96 kHz, NFFT = 1600, 50% overlap).

Eighteen whistles were classified into this group. These whistles exhibited contours with many local extrema and inflection points, had a high FM rate, and a long duration (Figs 4, 5, 6 and 7). Note the similarity of the overall upsweeping whistles in Figs 5, 6 and 7. These whistles are 1.1–1.2 s in duration, and have a fundamental contour starting at about 5 kHz and ending at about 8 kHz. The differences are that Fig 5 shows sidebands at the beginning, Fig 6 lacks overtones, and Fig 7 shows two similar whistles recorded almost simultaneously but with different FM rates.

#### Group BC02

**Fig 8:**
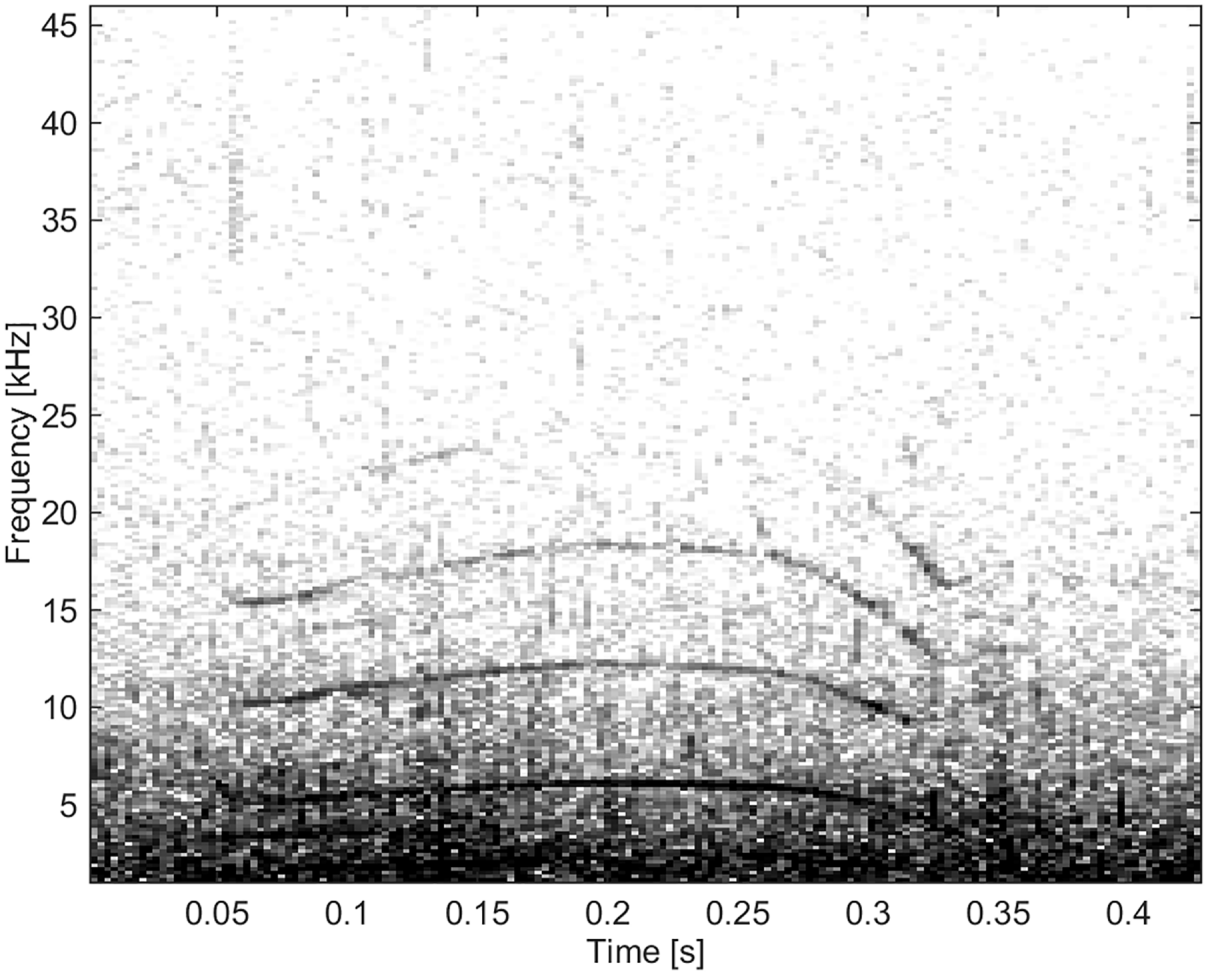
Whistle from Group BC02 with a short duration and convex shape. Harmonic overtones are present (fs = 96 kHz, NFFT = 512, 50% overlap).

**Fig 9:**
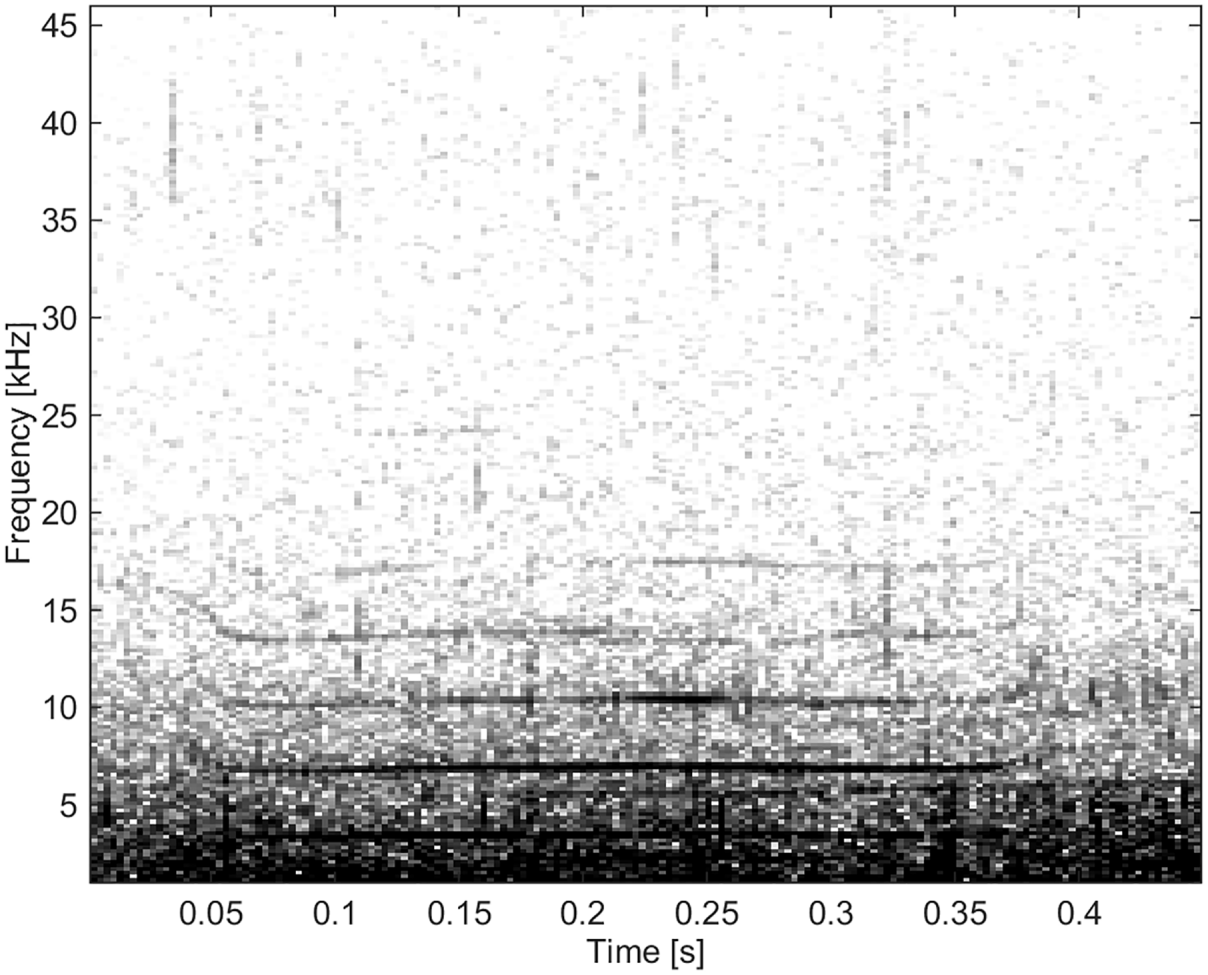
Whistle from Group BC02 of low frequency and a constant-wave shape. This whistle also has harmonic overtones (fs = 96 kHz, NFFT = 512, 50% overlap).

**Fig 10:**
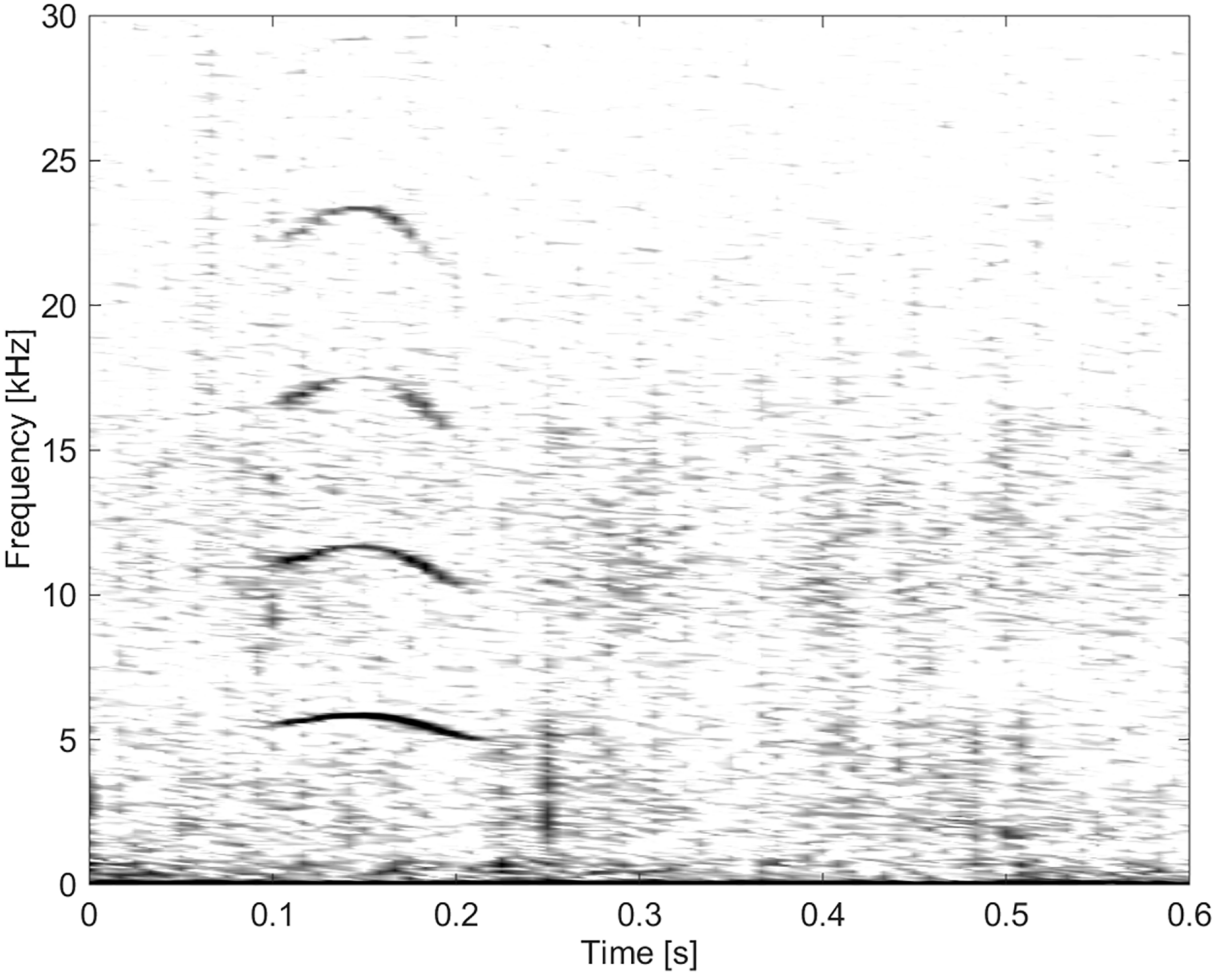
Whistle from Group BC02 of short duration and with a low number of extrema and inflection points (fs = 96 kHz, NFFT = 512, 50% overlap).

These whistles had the lowest frequencies and a low frequency range (Delta f). They were of short duration and had a low number of extrema and inflection points. Most calls, except three, had harmonic overtones at frequencies that were integer-multiples of the fundamental frequency. There were a total of 35 whistles categorized into this group (Figs 8, 9 and 10).

#### Group BC03

**Fig 11:**
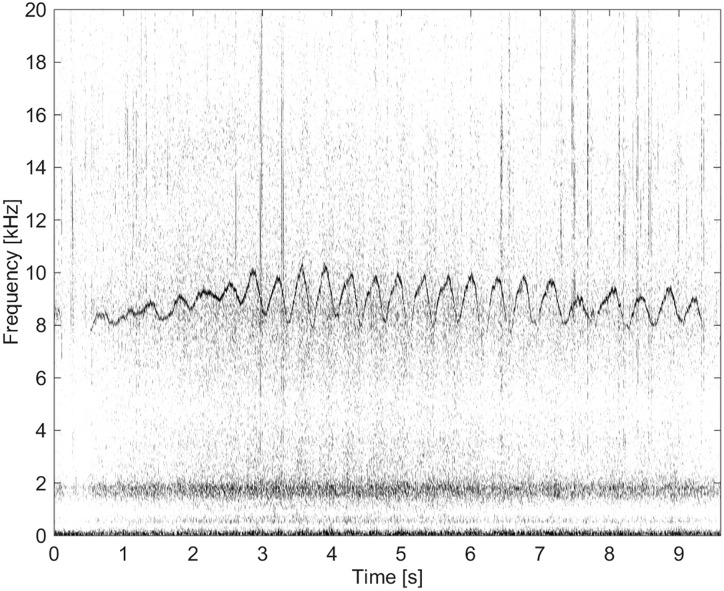
Whistle from Group BC03 with a long duration and a high number of extrema and inflection points (fs = 192 kHz, NFFT = 3200, 50% overlap).

**Fig 12:**
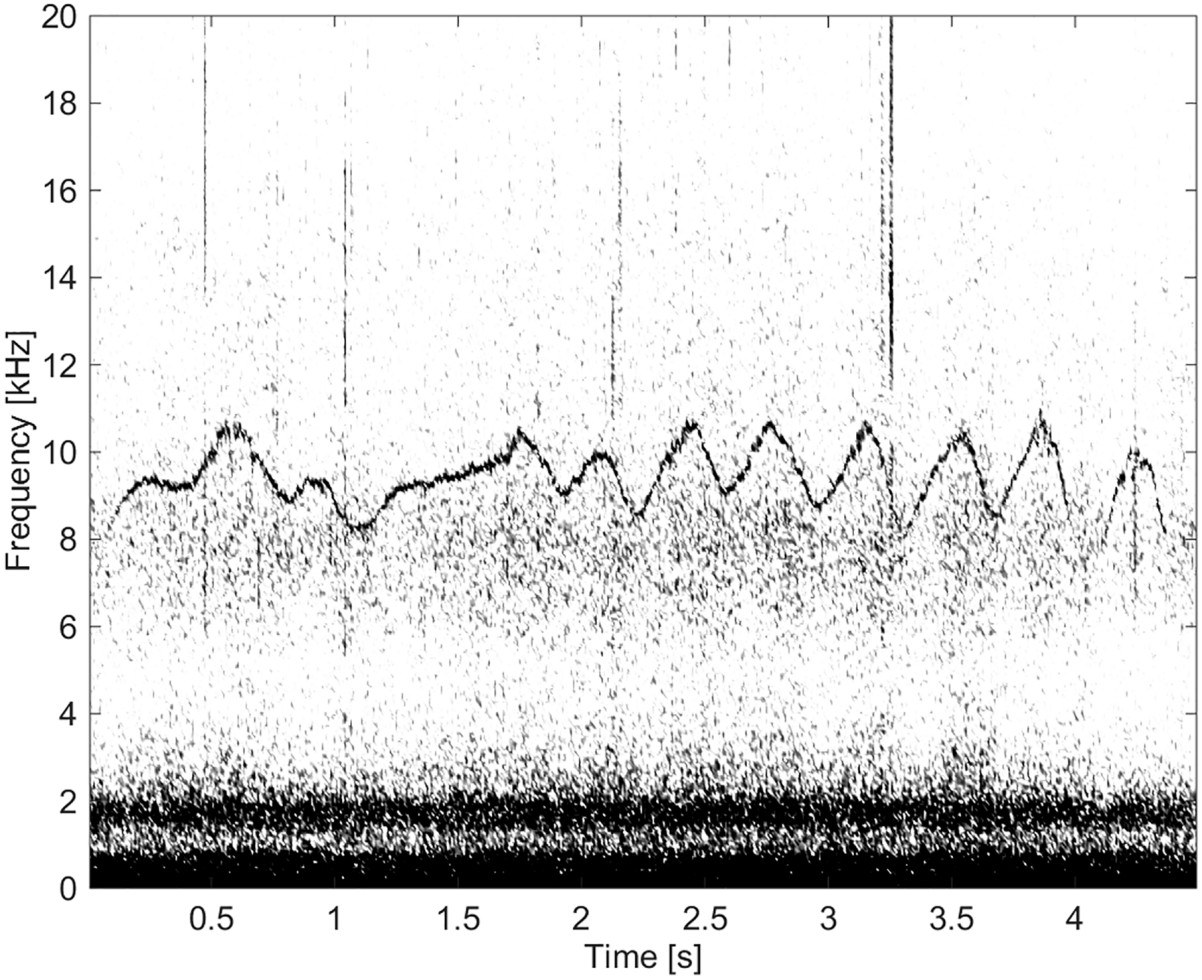
Whistle from Group BC03 with a long duration and a high number of extrema and inflection points (fs = 192 kHz, NFFT = 3200, 50% overlap).

This group comprised of five whistles. These whistles had the longest duration (11.3 s) and by far the highest number of extrema and inflection points (Figs 11 and 12). None of these whistles had overtones.

#### Group BC04

**Fig 13:**
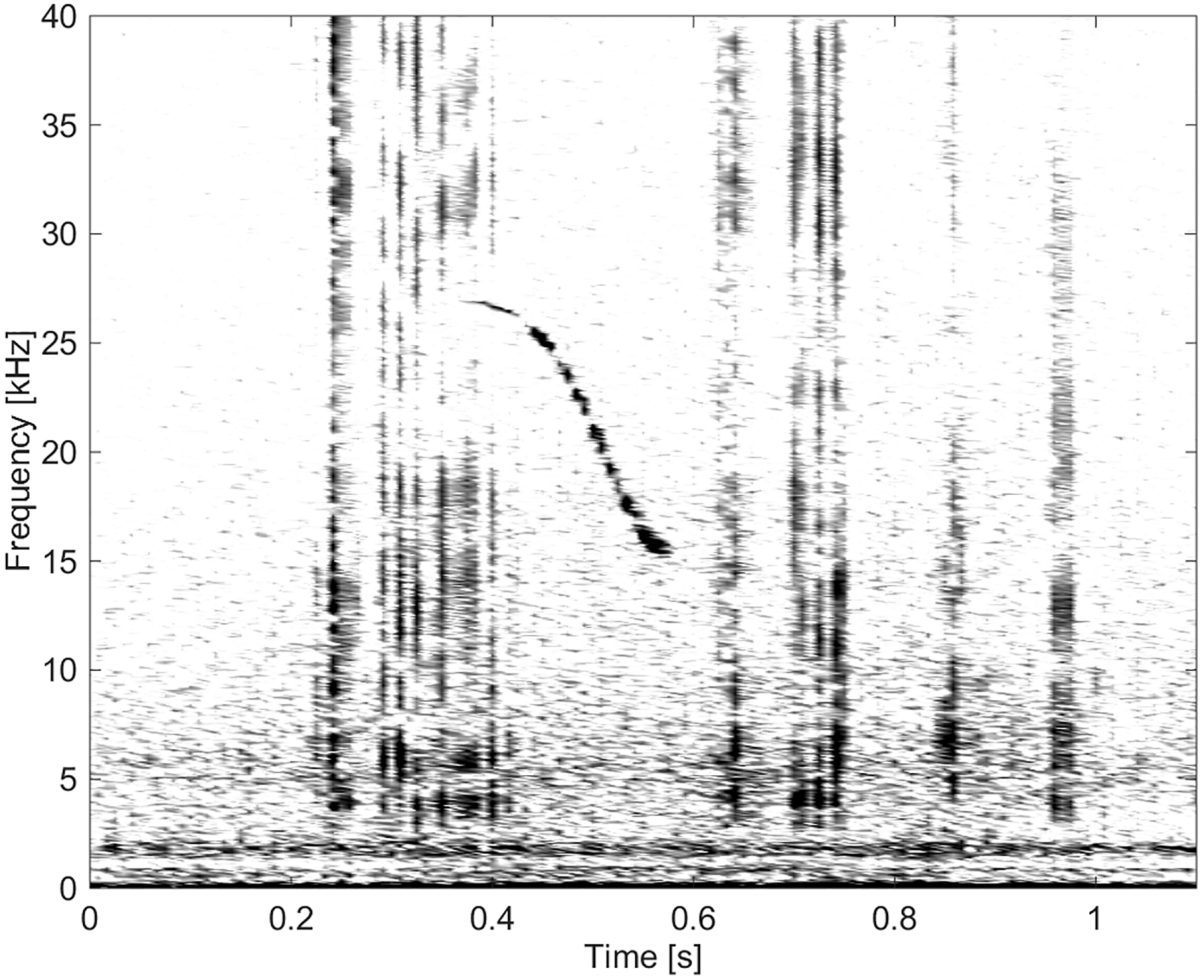
Whistle from Group BC04 of short duration and high frequency, ranging up to 27 kHz (fs = 192 kHz, NFFT = 3200, 50% overlap).

**Fig 14:**
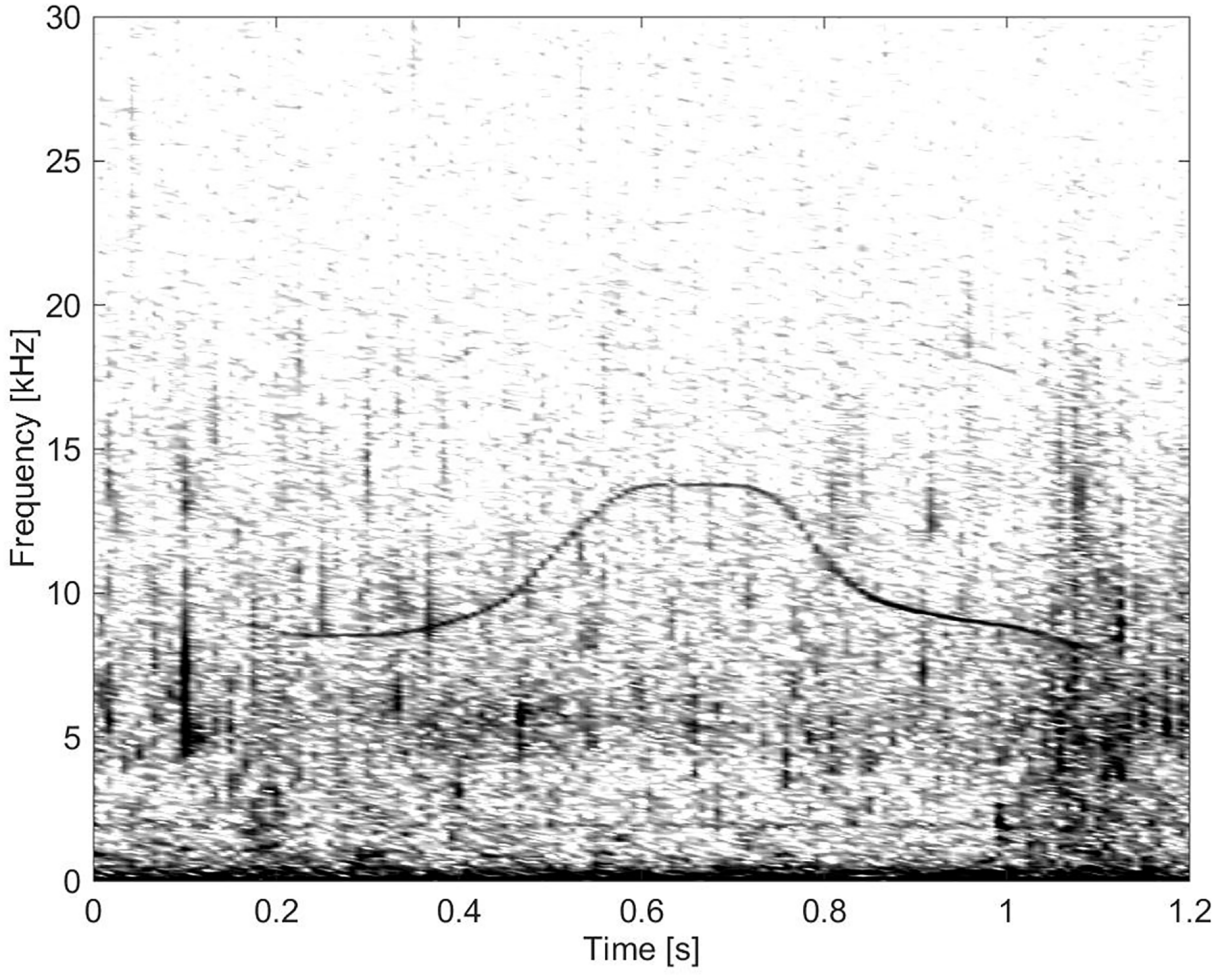
Whistle from Group BC04 demonstrating “simple” frequency modulation (fs = 96 kHz, NFFT = 1600, 50% overlap).

**Fig 15:**
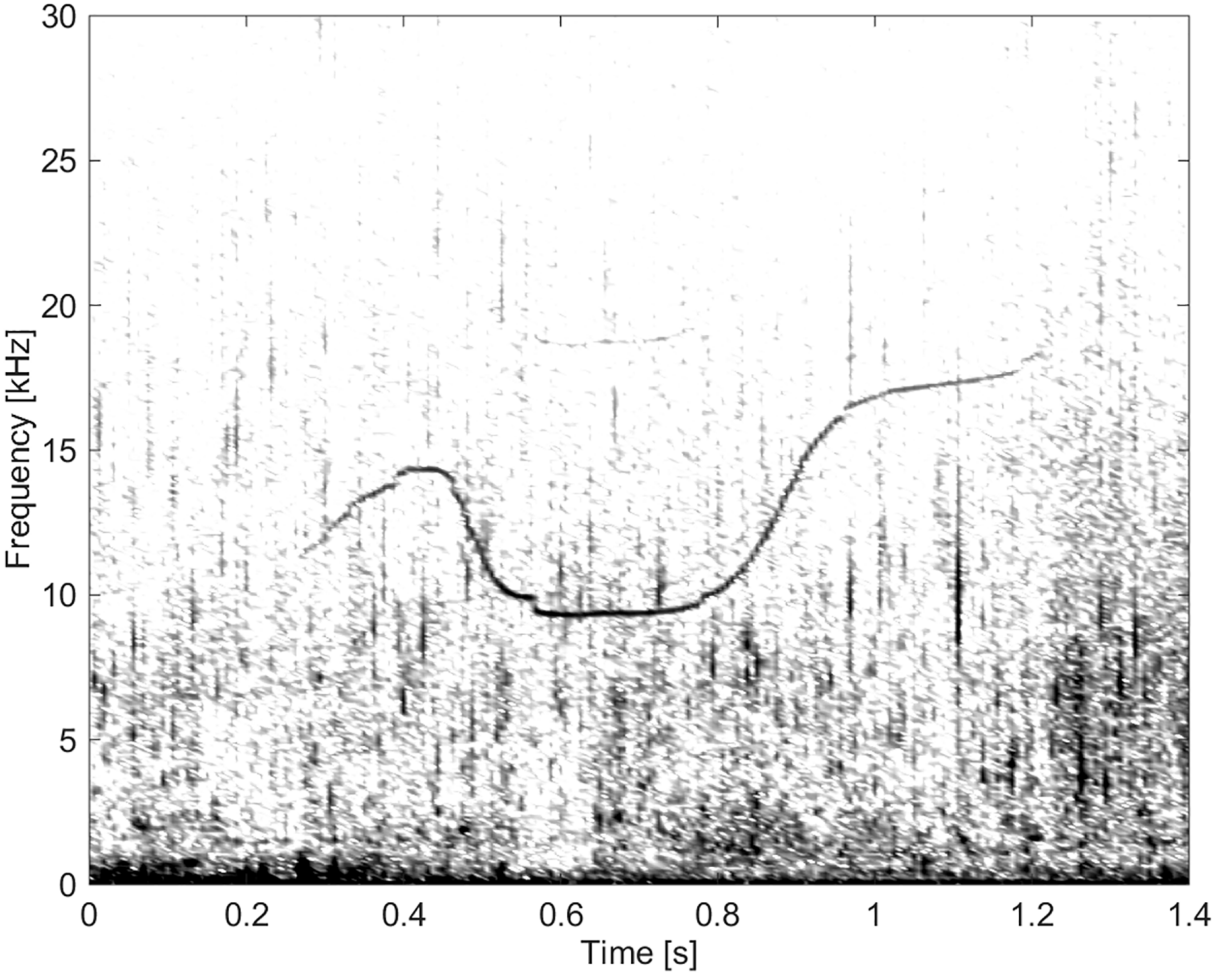
Whistle from Group BC04 demonstrating “simple” frequency modulation and high frequency, ranging up to 19 kHz (fs = 96 kHz, NFFT = 1600, 50% overlap).

These whistles were of high frequency and short duration with “simple” frequency-modulation and contours including upsweeps, downsweeps, concave and convex shapes (Figs 13, 14 and 15). There were a total of 61 whistles categorised into this group.

### Burst-pulse Sounds

#### Group BC05

**Fig 16:**
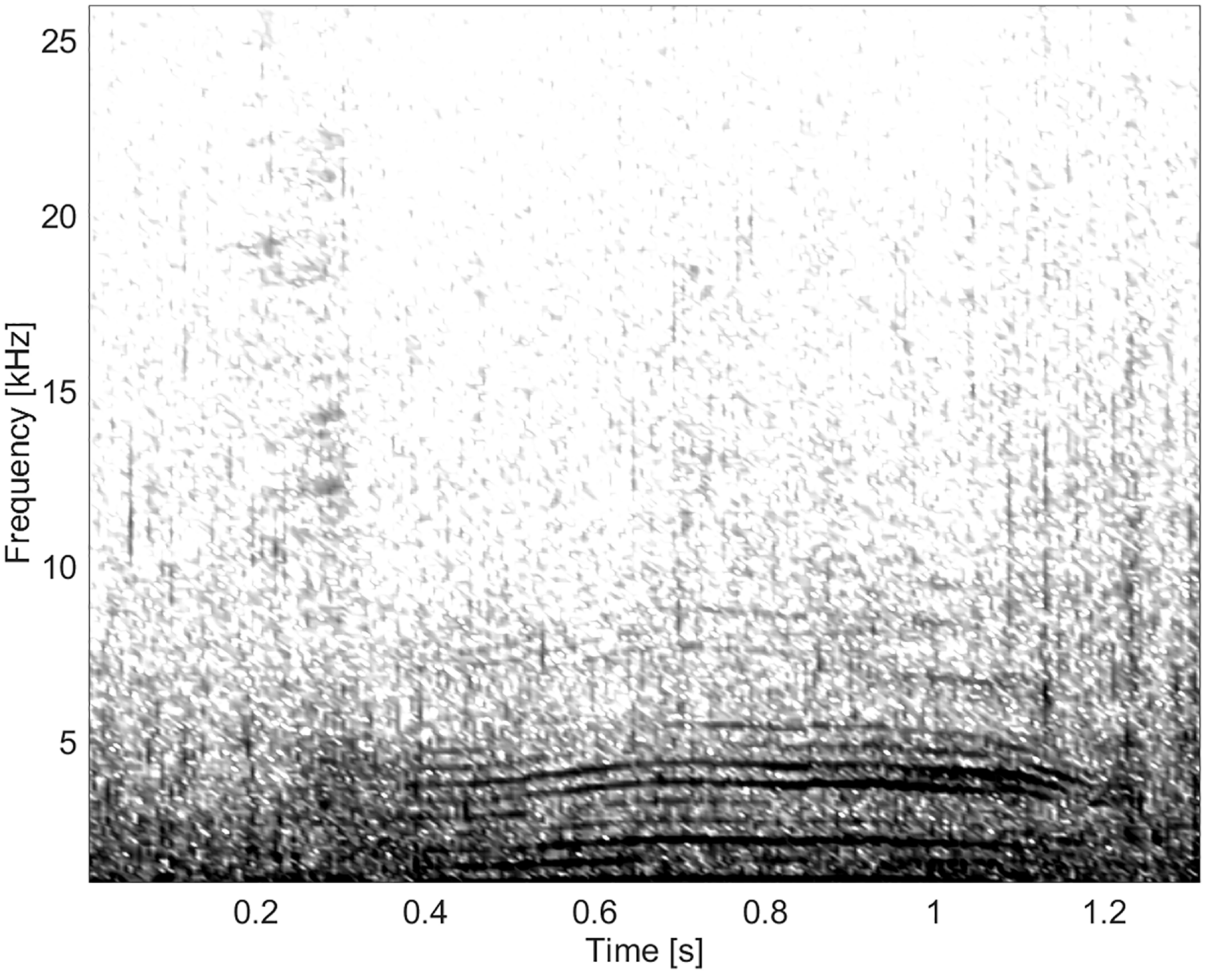
Burst-pulse sound from Group BC05 showing little frequency modulation (fs = 96 kHz, NFFT = 1024, 50% overlap).

This group consisted of five burst-pulse sounds with sidebands extending to the lowest frequencies, the lowest FM rates and the longest durations (Fig 16).

#### Group BC06

**Fig 17:**
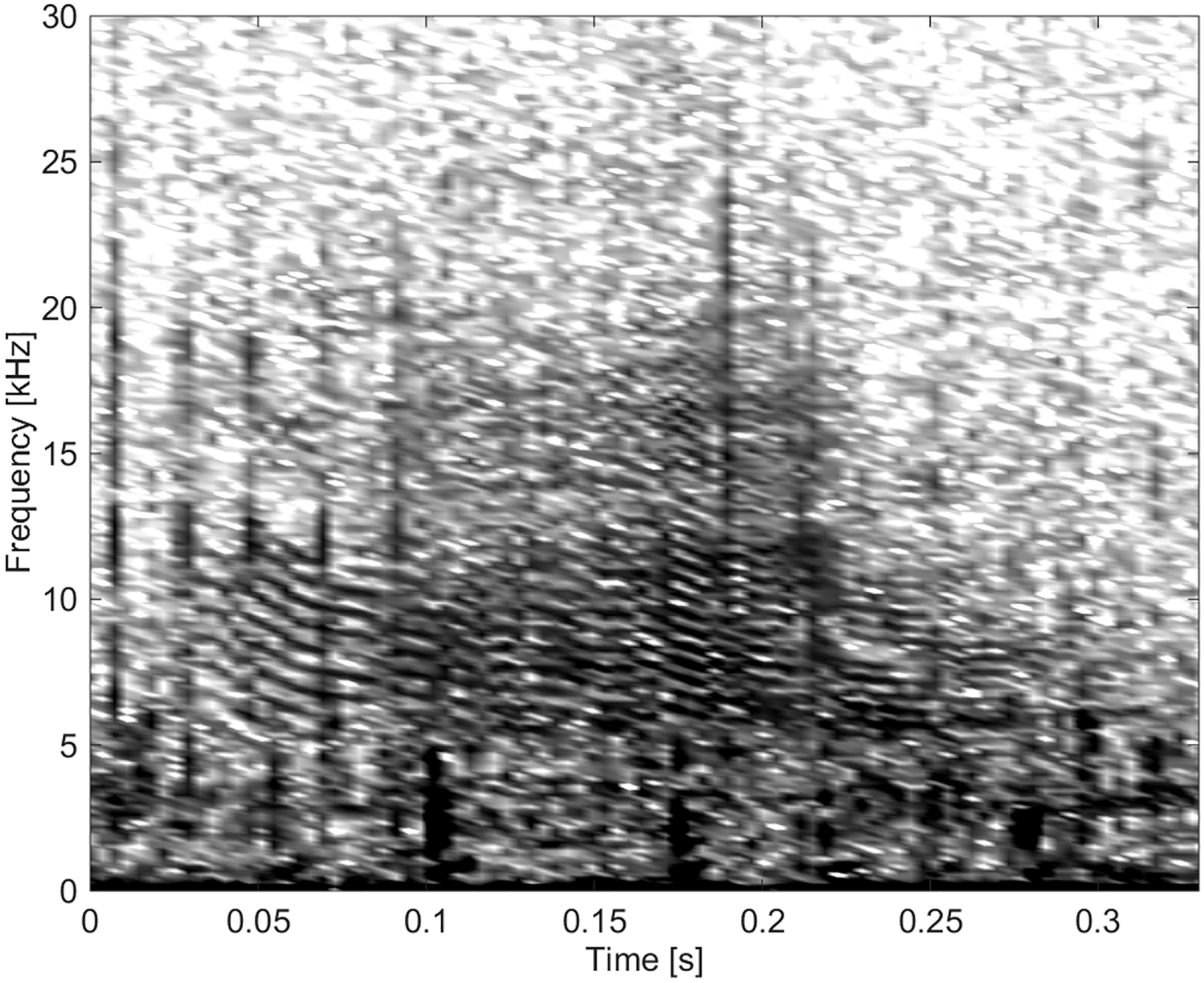
Burst-pulse sound from Group BC06 of high frequency, and with a high FM rate and many inflection points (fs = 96 kHz, NFFT = 700, 50% overlap).

These burst-pulse sounds had the highest frequencies, the highest number of extrema and inflection points, and the highest FM rates. There were a total of six calls categorized into this group (Fig 17).

#### Group BC07

**Fig 18:**
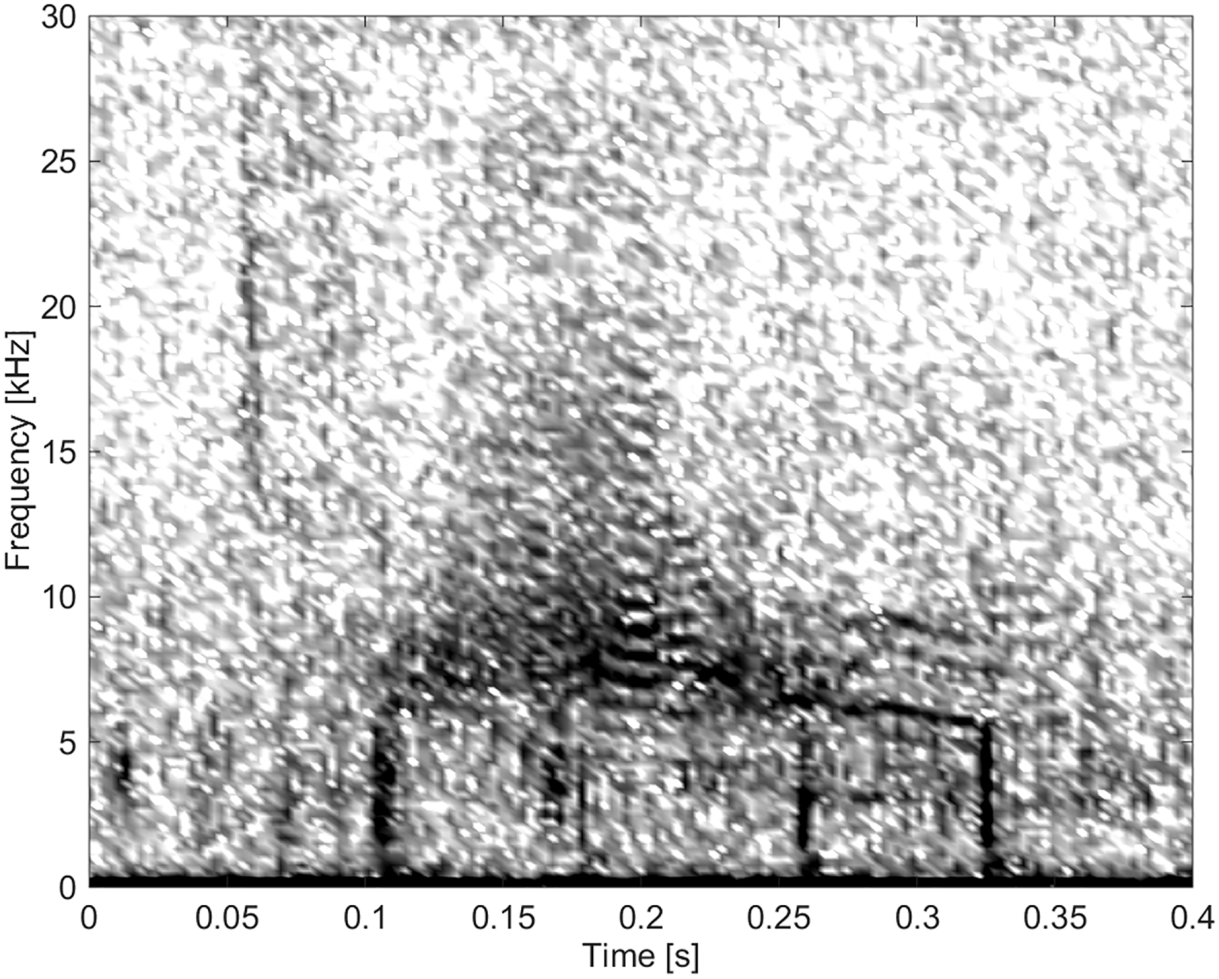
Burst-pulse sound from Group BC07 of short duration (fs = 96 kHz, NFFT = 512, 50% overlap).

These burst-pulse sounds were intermediate in frequency and the shortest in duration. There were a total of four calls categorised into this group (Fig 18).

#### Group BC08

**Fig 19:**
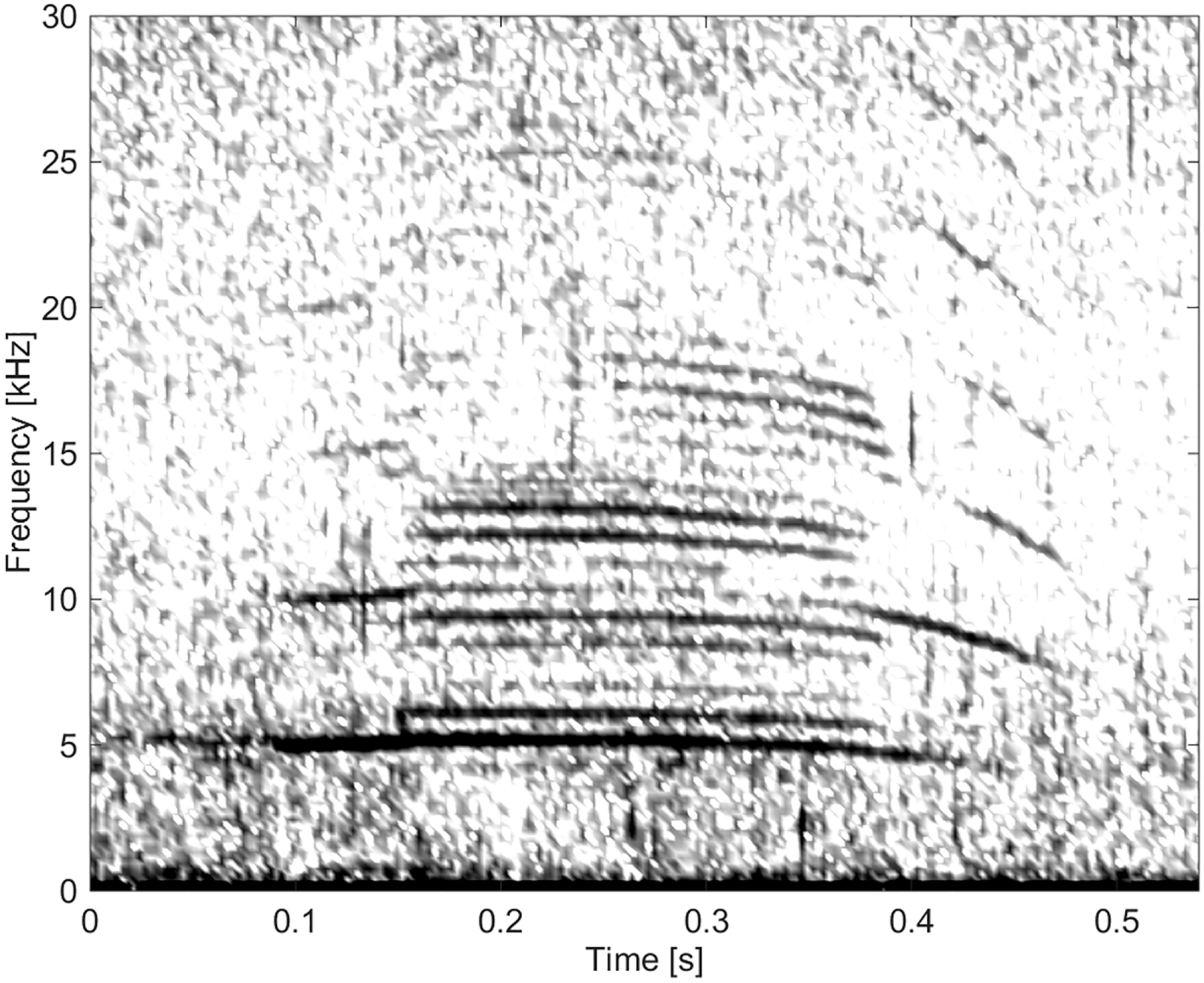
Call type BC08- a whistle that is pulsed in the middle- with non-harmonic sidebands only in the centre of the call (fs = 96 kHz, NFFT = 512, 50% overlap).

Group BC08 were whistles that were pulsed in the middle, hence exhibiting many sidebands only in the centre of the call, and harmonically-related overtones at the beginning and end of the call. This call type was recorded twice, once in a convex shape (Fig 19) and once in a concave shape.

#### Group BC09

**Fig 20:**
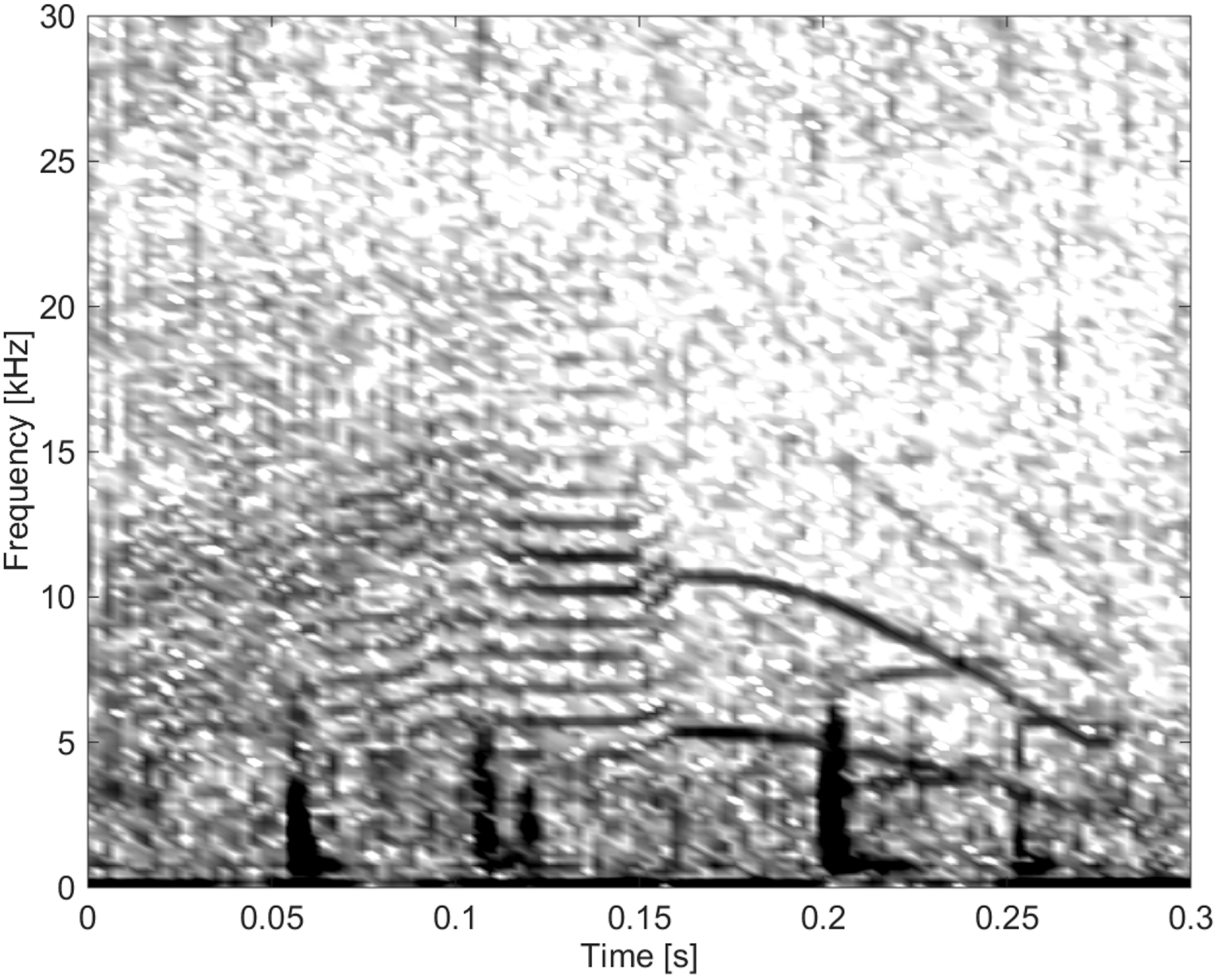
Call type BC09, a transition of burst-pulse sound with many sidebands changing into a frequency-modulated downsweep whistle with harmonic overtones (fs = 96 kHz, NFFT = 512, 50% overlap).

Group BC09 was recorded six times and consisted of both burst-pulse to whistle transitions and whistle to burst-pulse transitions. Calls had a duration of 0.1 to 1.2 s, with half of the call being of burst-pulse nature and the other half a whistle (Fig 20).

### Clicks

**Fig 21:**
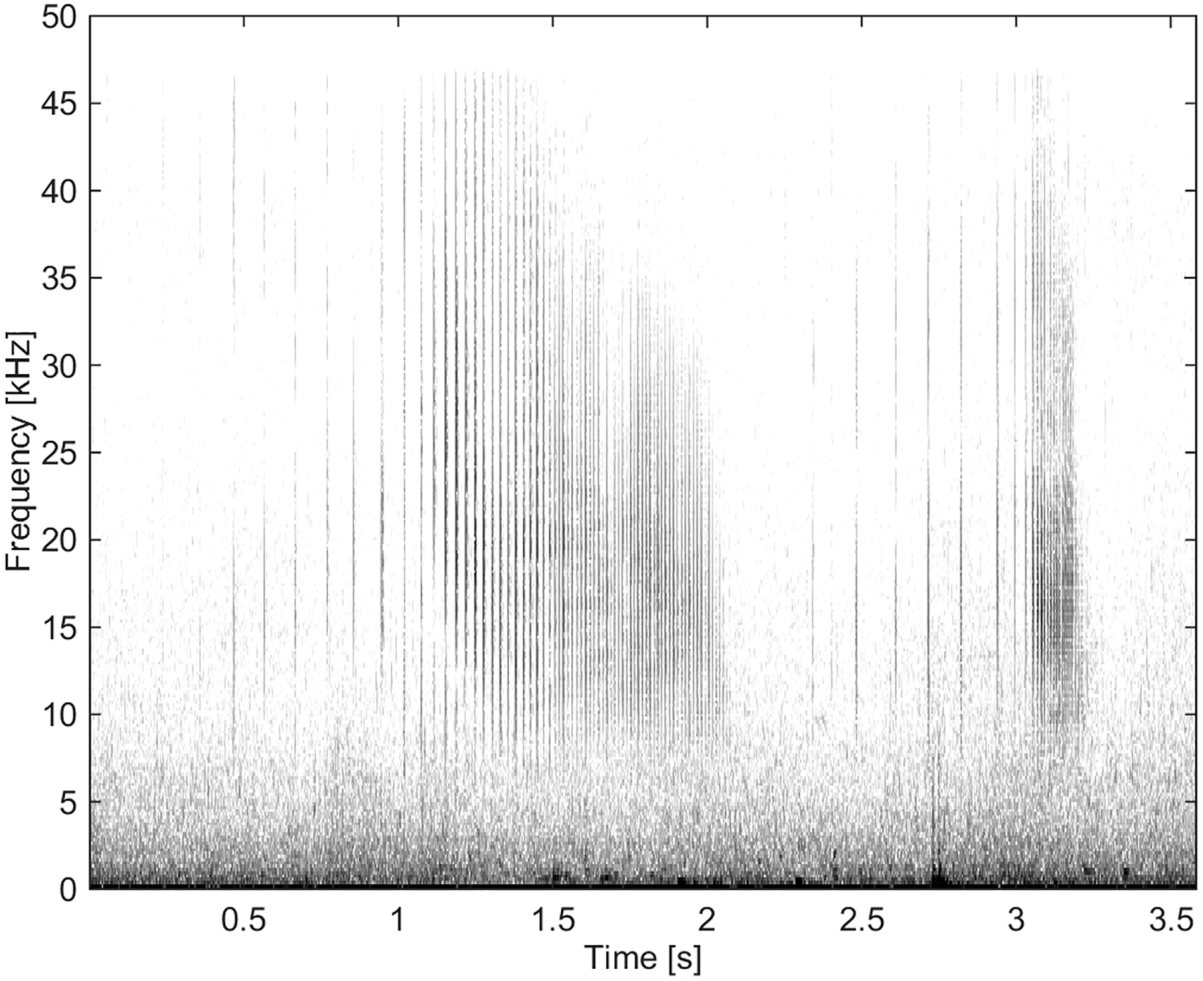
Two sped-up click trains with peak energy between 12 and 23 kHz (fs = 96 kHz, NFFT = 1600, 50% overlap).

**Fig 22:**
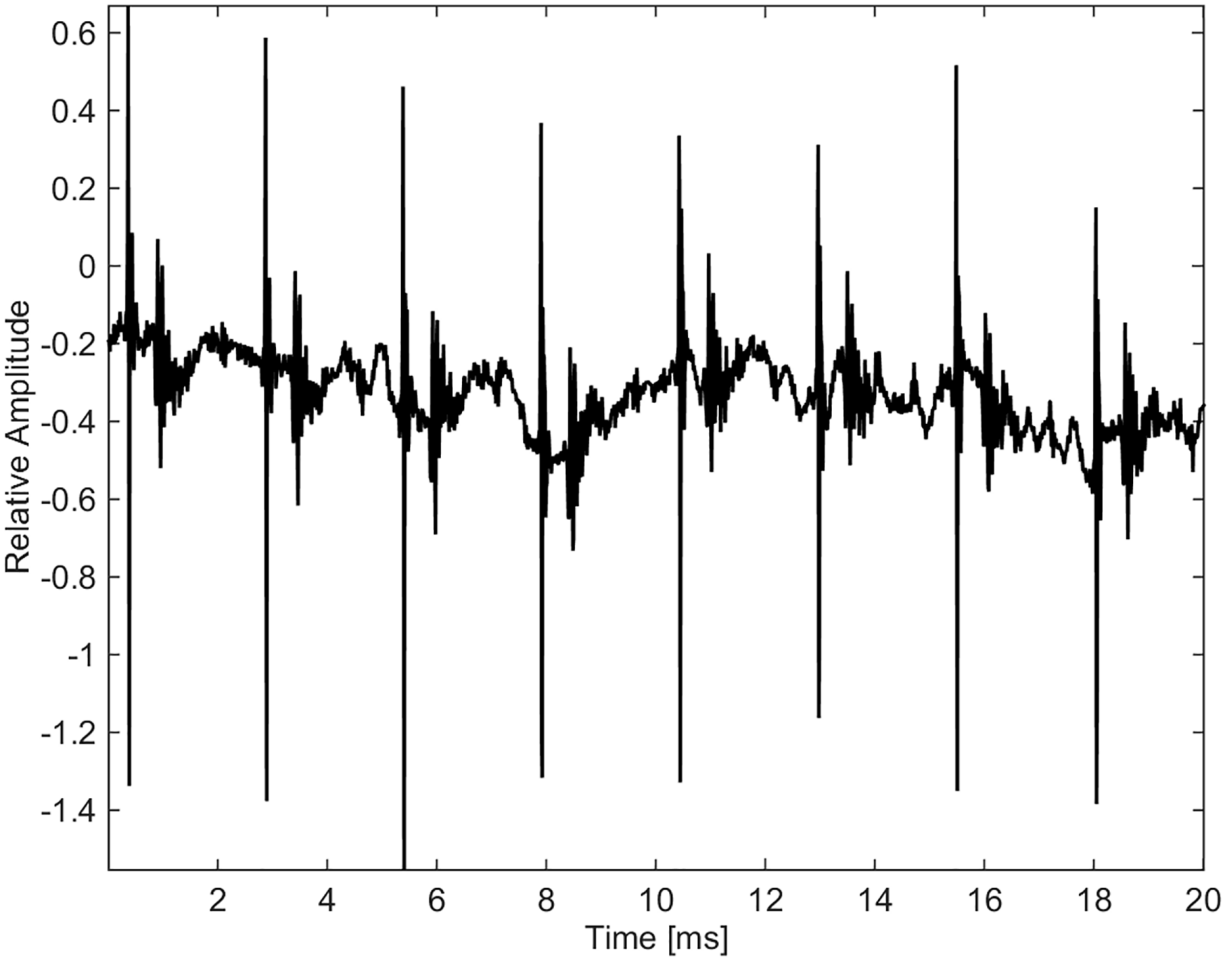
Buzz with inter-click interval of 2 ms and reflections seen 0.4 ms after the clicks (fs = 96 kHz).

**Fig 23:**
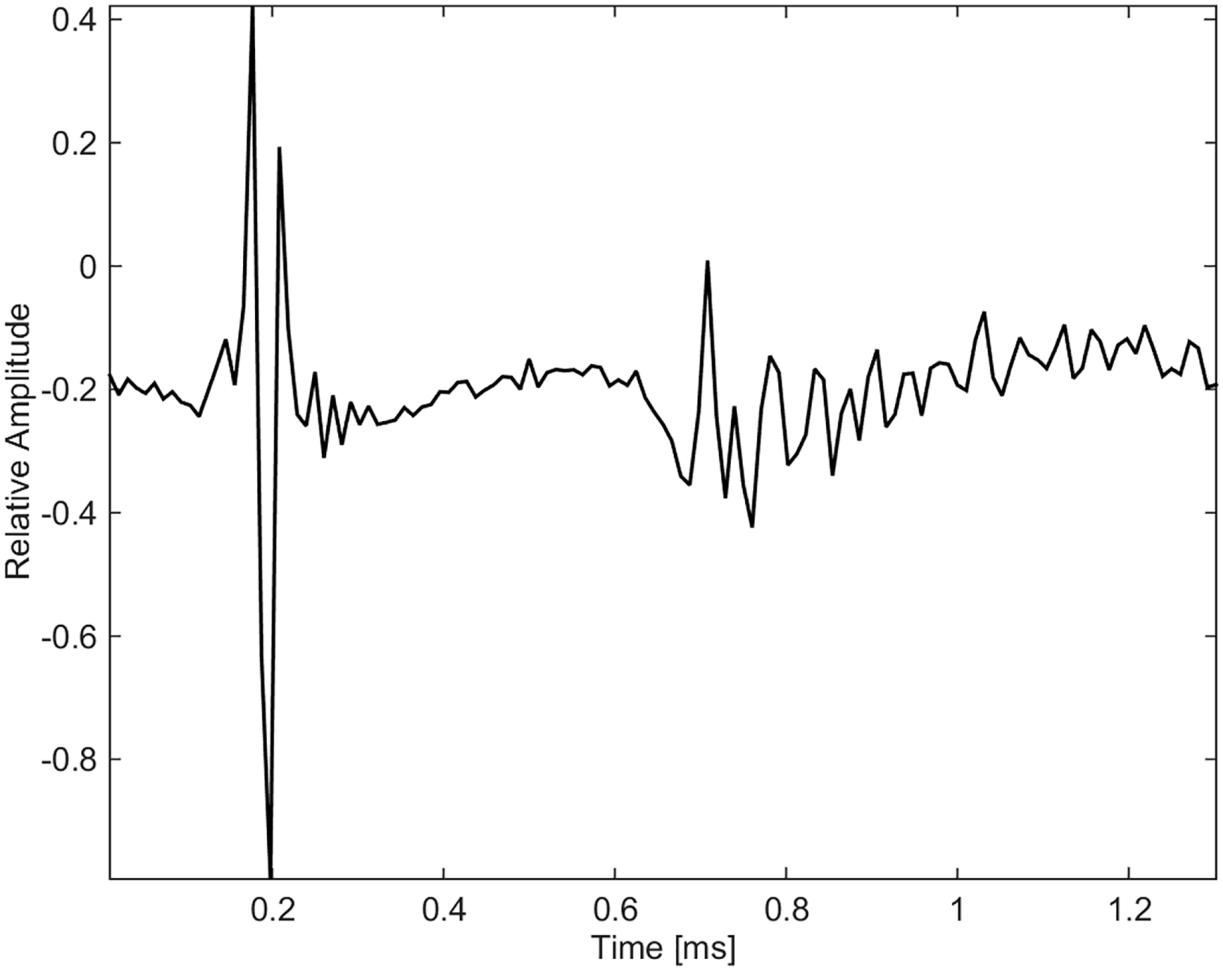
Single click of <200 μs duration in the shape of a negative Gabor function, with the reflection arriving 0.4 ms later (fs = 96 kHz).

Clicks were grouped collectively and separate from whistles and burst-pulse calls (Figs 21, 22 and 23). Some clicks were recorded as slow trains with inter-click intervals of 0.1 s, and sped-up ending in a buzz sound (Fig 21). The peak energy for clicks was between 12 and 24 kHz. Spectra and waveforms compare to those recorded from North Pacific killer whales [24]. Fig 22 shows a few clicks from a buzz sequence. The inter-click interval is 2.5 ms. Reflections are seen 0.5 ms after each click. Fig 23 is a zoomed-in version of Fig 22, showing the Gabor waveform of an outgoing click likely recorded on-axis, i.e. the animal was echolocating in the direction of the hydrophone.

## Discussion

The Australian killer whale vocalisations analysed in this study demonstrate a repertoire of whistles, pulsed calls, and echolocation clicks, similar to those reported from killer whales in other regions. While biosonar clicks are not expected to vary between populations, and have not been used in the literature to distinguish between populations, the whistles and burst-pulse sounds characterised here provide a basis for initial comparison to other populations worldwide. Nine call types were categorised and grouped accordingly in this analysis.

Comparing our call types to the only other killer whale calls reported from the southern hemisphere, two whistles categorised in BC01 were strikingly similar to call type AM4 recorded in Antarctica [40]. Both vocalisations are overall upsweeping whistles of 1–1.2 s in length, fundamentals of 4–7 kHz plus harmonics, and with many inflection points (Figs 5 and 7). Some of the vocalisations in our group BC02 were very similar to AM2 [40], with whistles of 0.2–0.4 s duration, fundamentals of 4–7 kHz plus harmonics, with hardly any frequency-modulation (Fig 10). Our call type Group BC09 which consisted of whistle and burst-pulse transitioning calls, was similar to call type AM5 [40], a buzz sequence which graded into a down-sweeping frequency-modulated signal rich in harmonics. We did not record any of the other four call types documented for Antarctic killer whales [40].

Interestingly, whistle maximum frequency appears to vary substantially across killer whale populations, in contrast to what is reported for other delphinids [53]. Whistle fundamental frequencies have been reported up to 36 kHz in the North Pacific region [23, 28–31] and up to 74 kHz in Norwegian and Icelandic killer whales [32]. Our research shows Australian killer whales exhibit whistle frequencies well within the range of documented bandwidths across other regions, with whistle fundamental frequencies ranging up to 29 kHz.

While 34% of our recorded calls had zero inflection points, the FM rate, i.e. the mean number of inflection points per second of call, was 5.9 / s (median 2.9 / s). Some of our calls had very high numbers of inflections per second (peak 50 / s). High FM rates were also noted for Antarctic killer whales with a mean of 7.5 / s, median 8.4 / s, computed as the ratio of mean number of inflections and mean duration for all call types listed in Table 1 of [40]. Resident killer whales off Vancouver Island, British Columbia, have been reported to show high FM rates of up to 20/s [23], often sped-up variants of slower calls. These ‘excitement calls’ were recorded during episodes of physical interactions between animals both at the surface and underwater as observed by Ford [23].

Many of the Pacific northwest resident killer whale calls consist of several parts with different frequency content and modulation [39]. Calls categorised in BC09 were both burst-pulse to whistle transitions and vice versa. These calls typically consisted of two parts, either beginning with the whistle or the burst-pulse component, and then transitioning to the other component.

This study provides the foundation to continue further analysis and comparison, and has depicted some basic signal structure characteristics found in the Australian killer whale population.

In addition to comparing Australian killer whale sounds to other populations worldwide, further investigation and comparison of killer whale populations found within Australian waters could greatly benefit our limited knowledge of this species in this region, with the ability to uncover potential distinctive acoustic repertoires and possible sympatric ecotypes in Australia.

Previous studies of the vocal behaviour of different killer whale populations have revealed quantitative and qualitative differences related to dietary specialisation. In the north-western Pacific, mammal-hunting killer whales have been shown to produce echolocation clicks, pulsed calls and whistles at significantly lower rates compared to sympatric fish eaters [4, 29, 54]. Whereas many fish species have poor hearing sensitivity at the frequencies of killer whale vocalisations, marine mammals can detect killer whale vocalisations at significant distances, and this eavesdropping from their potential prey makes vocal behaviour costly for mammal hunting killer whales [4, 55]. Therefore, since acoustic behaviour can be a tool for indicating foraging specialists, the analysis of Australian killer whale sounds might determine acoustic differences and geographic variations associated with different foraging strategies, and potential undescribed different ecotypes in this region.

Obtaining a detailed description of the acoustic characteristics of killer whales in Australia is necessary for species acoustic identification, and allows us to use passive acoustic monitoring as a tool for monitoring the population. Passive acoustic monitoring is potentially a powerful, non-lethal, non-invasive and cost-effective method for assessing killer whale abundance and trends, defining habitat use and monitoring population. This would further enhance our limited knowledge and gain an understanding of both spatial and temporal distribution of killer whales in Australian waters.
