# Correction: Vocalisations of Killer Whales (*Orcinus orca*) in the Bremer Canyon, Western Australia

**DOI:** 10.1371/journal.pone.0140738

**Published:** 2015-10-13

**Authors:** Rebecca Wellard, Christine Erbe, Leila Fouda, Michelle Blewitt

There are errors in the Funding section. The correct funding information is as follows: The 2015 data collection was supported by the SeaWorld and Busch Gardens Conservation Fund (http://swbg-conservationfund.org/). Authors who received the funding were CE and LF. The funders had no role in study design, data collection and analysis, decision to publish, or preparation of the manuscript.
